# Oxcarbazepine-induced tardive dyskinesia: A rare adverse reaction

**DOI:** 10.4103/1817-1745.66664

**Published:** 2010

**Authors:** M. Özlem Hergüner, Faruk Incecik, Şakir Altunbaşak

**Affiliations:** Department of Pediatric Neurology, Cukurova University Faculty of Medicine, Adana, Turkey

Sir,

Epilepsy is characterized by recurrent unprovoked seizures and is one of the most prevalent neurologic diseases. As a result of the limitations of older drugs and improvements in our understanding of epilepsy and the mechanisms of action of the drugs used to treat it, several new agents have been approved in the past decade.

Oxcarbazepine (OXC) is a new anti-epileptic drug that has been registered in several countries worldwide since 1990.[1] It is a keto analog of carbamazepine and has a more favorable pharmacokinetic profile. The results of clinical trials suggest that it is better tolerated than carbamazepine.[1–3] The most common adverse events are usually related to the central nervous (dizziness, diplopia, ataxia, headache, weakness, nystagmus, slurred speech) or gastrointestinal systems (nausea, vomiting, epigastric distress, diarrhea). The other adverse events are allergic rashes and hyponatremia.

We now report a child who experienced tardive dyskinesia soon after OXC therapy.

An 8-year-old girl was admitted to the hospital with two complex partial seizures in the last month. She was born from nonconsanguineous marriage with uncomplicated delivery. Her mental and motor development were normal. Her physical and neurological examinations were also normal. In history, she was diagnosed as attention-deficit hyperactivity disorder (ADHD) 1 year ago at the pediatric psychiatry outpatient clinic, without medical treatment.

Magnetic resonance imaging revealed no abnormality. Interictal electroencephalogram showed rolandic spikes in the left hemisphere with normal background activity. OXC therapy was begun at 15 mg/kg daily for 1 week and then 30 mg/kg/d. Three days after full-dose treatment, she was admitted to the emergency service with abnormal movements. In her examination, she had trismus, tongue protrusion, deviation of the eyes and lateral flexion of the trunk. She was diagnosed as tardive dyskinesia. OXC was stopped. Single-dose intravenous diazepam was administered and then oral difenhydramine treatment was given for 2 weeks. Her symptoms ceased in 3 days.

Acute dystonic reactions occur after exposure to dopamine (DA) receptor blocking agents, a class that includes neuroleptic agents and antiemetic agents. It is well recognized that some anticonvulsant therapy may also be associated with various dyskinesias, including chorea, choreoathetosis, dystonia and asterixis. Phenitoin, primidone, phenobabitone, phenobarbital, ethosuximide and carbamazepine have been implicated as offending agents.[4,5] Dyskinesias due to anticonvulsants usually result from toxicity and initial exposure of drug or, more commonly, during chronic usage.

Tardive dyskinesia is not a common adverse reaction after OXC treatment. In the literature, there are rare reports about carbamazepine-induced tic disorders and tardive dyskinesia-like syndrome.[6,7] It was thought that the presence of striatal DA receptor supersensitivity serves as the prevailing hypothesis regarding the underlying neurobiological mechanism for tics.[6] This is based on the following observations: DA receptor antagonists are the most effective drugs for suppressing tics and that tics may be worsened by drugs that enhance dopaminergic neurotransmission, such as amphetamins. This dopaminergic effect of carbamazepine may be responsible for the induction of tics, but the drug has a multiplicity of other central neurochemical effects that may be involved as well. The possible mechanism of OXC-induced dyskinesia may be similar to that of carbamazepine.

Although there are conflicting results in the literature, carbamazepine has been reported to be useful in the treatment of tardive dyskinesia related to chronic neuroleptic use.[8,9] But, it is not clear, whether it is the result of a state-dependent condition rather than the action of carbamazepine.

ADHD is a neurobehavioral syndrome, both defined and diagnosed by the presence of a certain number and intensity of behaviors classified into three symptom groups: inattention, impulsivity and hyperactivity. In ADHD pathogenesis, besides various endogen and exogen factors, some abnormalities in the central dopaminergic system have been reported. In these patients, the level of homovalinic acid that is the principal metabolite of DA has been found to be abnormal.[10] Because of this, it is important to be careful when using agents that induced the dopaminergic system in these patients.

In conclusion, tardive dyskinesia is a rare adverse reaction during OXC therapy, also seen in the early phase of therapy. Use of the Naranjo ADR Probability Scale indicated a probable (the score was 6) relationship between tardive dyskinesia and OXC therapy in this patient.[11] Such agents that affect the central dopaminergic systems should be administered with caution in patients having dopaminergic abnormalities.
